# Treadmill walking economy is not affected by body fat and body mass index in adults

**DOI:** 10.14814/phy2.16023

**Published:** 2024-05-17

**Authors:** Wesley J. Tucker, Brandon J. Sawyer, Dharini M. Bhammar, Emma W. Ware, Siddhartha S. Angadi, Glenn A. Gaesser

**Affiliations:** ^1^ Department of Nutrition & Food Sciences Texas Woman's University Houston Texas USA; ^2^ Institute for Women's Health, College of Health Sciences Houston Texas USA; ^3^ Department of Kinesiology & Department of Biology Point Loma Nazarene University San Diego California USA; ^4^ Center for Tobacco Research, Division of Medical Oncology, Department of Internal Medicine The Ohio State University Columbus Ohio USA; ^5^ Department of Kinesiology, School of Education and Human Development University of Virginia Charlottesville Virginia USA; ^6^ College of Health Solutions Arizona State University Phoenix Arizona USA

**Keywords:** adiposity, body mass index, energy cost of walking, metabolic rate, obesity

## Abstract

To determine whether body fat and body mass index (BMI) affect the energy cost of walking (Cw; J/kg/m), ventilation, and gas exchange data from 205 adults (115 females; percent body fat range = 3.0%–52.8%; BMI range = 17.5–43.2 kg/m^2^) were obtained at rest and during treadmill walking at 1.34 m/s to calculate gross and net Cw. Linear regression was used to assess relationships between body composition indices, Cw, and standing metabolic rate (SMR). Unpaired *t*‐tests were used to assess differences between sex, and one‐way ANOVA was used to assess differences by BMI categories: normal weight, <25.0 kg/m^2^; overweight, 25.0–29.9 km/m^2^; and obese, ≥30 kg/m^2^. Net Cw was not related to body fat percent, fat mass, or BMI (all *R* ^2^ ≤ 0.011). Furthermore, mean net Cw was similar by sex (male: 2.19 ± 0.30 J/kg/m; female: 2.24 ± 0.37 J/kg/m, *p* = 0.35) and across BMI categories (normal weight: 2.23 ± 0.36 J/kg/m; overweight: 2.18 ± 0.33 J/kg/m; obese: 2.26 ± 0.31, *p* = 0.54). Gross Cw and SMR were inversely associated with percent body fat, fat mass, and BMI (all *R* ^2^ between 0.033 and 0.270; all *p* ≤ 0.008). In conclusion, Net Cw is not influenced by body fat percentage, total body fat, and BMI and does not differ by sex.

## 1 INTRODUCTION

Walking is a popular mode of exercise. For individuals with obesity, greater body mass may increase metabolic rate such that exercise oxygen uptake (V̇O_2_)—expressed in absolute terms, unadjusted for body mass—at any given speed of walking is increased when compared with individuals without obesity (Browning et al., 2006; James et al., 1978). However, published data on the influence of body fat and body mass index (BMI; kg/m^2^) on walking economy, expressed as either metabolic rate (W/kg) or energy cost per distance walked (Cw; J/kg/m), have yielded differing conclusions (Bode et al., 2020; Browning et al., 2006, 2013; Browning & Kram, 2005; Farrell et al., 1985; Fernandez Menendez et al., 2019, 2020; Lafortuna et al., 2008; Laroche et al., 2015; LeCheminant et al., 2009; Peyrot et al., 2009; Primavesi et al., 2021) (Table 1). A higher net metabolic rate during walking (standing W/kg subtracted from gross W/kg) has been reported for adults with obesity (Browning et al., 2006; Browning & Kram, 2005), and a higher net Cw during walking has been reported in both adolescents (Peyrot et al., 2009) and adults (Fernandez Menendez et al., 2019, 2020; Primavesi et al., 2021) with obesity. These studies indicated that net W/kg or J/kg/m were 10%–45% higher in individuals with obesity. Moreover, previously published regression analyses indicated that net W/kg during walking was positively related to percent body fat in adults (Browning et al., 2006) and to body mass in females (Lafortuna et al., 2008). In contrast to these studies, net W/kg (Browning et al., 2013; Lafortuna et al., 2008; LeCheminant et al., 2009), net Cw (Bode et al., 2020; Fernandez Menendez et al., 2019), and gross Cw (Browning et al., 2006; Browning & Kram, 2005) during walking have also been reported to be similar between adults with or without obesity. Furthermore, one study reported that net V̇O_2_ (mL/kg/min) during walking was lower in females with a mean BMI of 48 kg/m^2^ compared to females with a mean BMI of 22.2 kg/m^2^ (Farrell et al., 1985).

**TABLE 1:** Results of prior published papers on body mass index (BMI), percent body fat, and walking economy in adults.

Study	Participants	Walking speeds	Steady‐state verified?	Major findings (higher vs. lower BMI)
Browning and Kram (2005)	20 Females BMI: 20.4 ± 2.1 kg/m^2^ (*n* = 10) BMI: 34.1 ± 3.2 kg/m^2^ (*n* = 10)	0.5, 0.75, 1.25, 1.5, 1.75 m/s	Yes (no change in V̇O_2_ during last 2 min of each walking speed)	Greater net W/kg in higher BMI group Gross Cw not different between groups
Browning et al. (2006)	20 males BMI: 22.3 ± 1.9 kg/m^2^ (*n* = 10) BMI: 33.5 ± 2.1 kg/m^2^ (*n* = 10) 19 females BMI: 20.4 ± 2.1 kg/ m^2^ (*n* = 10) BMI: 33.8 ± 3.3 kg/ m^2^ (*n* = 9)	0.5, 0.75, 1.25, 1.5, 1.75 m/s	Not reported	Greater net W/kg in higher BMI groups Gross Cw not different across all groups Net W/kg positively correlated with percent body fat (*r* ^2^ = 0.43; *p* < 0.001)
Browning et al. (2013)	23 males; 28 females BMI: 21.6 ± 2.0 kg/m^2^ (*n* = 19; 10 females) BMI: 33.9 ± 3.5 kg/m^2^ (*n* = 32; 18 females)	1.25, 1.5, 1.75 m/s	Yes (no change in V̇O_2_ during last 2 min of each walking speed)	Net W/kg not different between groups Lower gross W/kg in higher BMI group
Bode et al. (2020)	6 males; 14 females BMI: 24.2 ± 1.3 kg/m^2^ (*n* = 10; 7 females) BMI: 33.1 ± 2.0 kg/m^2^ (*n* = 10; 7 females)	1.25 m/s	Not reported	Gross Cw and net Cw not different between groups Gross Cw inversely correlated with BMI (*r* = −0.44; *p* < 0.008)
Laroche et al. (2015)	11 males; 15 females BMI: 22.4 ± 1.8 kg/m^2^ (*n* = 13; 8 females) BMI: 29.5 ± 4.1 kg/m^2^ (*n* = 13; 7 females)	0.83 m/s	“Assumed”	Gross Cw greater in higher BMI group Gross Cw positively correlated with percent body fat (*r* = 0.42; *p* = 0.018), fat mass (*r* = 0.58; *p* = 0.001), and BMI (*r* = 0.51; *p* = 0.005)
Farrell et al. (1985)	17 females %fat 23.4 ± 4.0; mean BMI: ~22.2 kg/m^2^ (*n* = 10) %fat 50.4 ± 7.1%; mean BMI: ~48.0 kg/m^2^ (*n* = 7)	1.25 m/s	Not reported	Net VO_2_ (ml/kg/min) lower in group with higher % fat and BMI
Fernandez Menendez et al. (2019)	9 males; 17 females BMI: 21.9 ± 1.5 kg/m^2^ (*n* = 13; 8 females) BMI: 33.8 ± 2.5 kg/m^2^ (*n* = 13; 9 females)	0.56, 0.83, 1.11, 1.39, 1.67 m/s	Yes (steady metabolic state with RER <1.0)	Net and gross Cw not different at all speeds except 1.67 m/s (greater in higher BMI group)
Fernandez Menendez et al. (2020)	7 males; 17 females BMI: 22.0 ± 1.5 kg/m^2^ (*n* = 13; 8 females) BMI: 43.0 ± 4.2 kg/m^2^ (*n* = 11; 9 females)	0.56, 0.83, 1.11, 1.39, 1.67 m/s	Yes (RER <1.0)	Net Cw greater in higher BMI group at all speeds
Primavesi et al. (2021)	11 males; 37 females BMI: 22.0 ± 1.5 kg/m^2^ (*n* = 13; 8 females) BMI: 32.2 ± 1.5 kg/m^2^ (*n* = 17; 15 females) BMI: 40.1 ± 4.4 kg/m^2^ (*n* = 18; 14 females)	0.56, 0.83, 1.11, 1.39, 1.67 m/s	Yes	Net Cw greater in highest BMI group compared to lowest BMI group Net Cw not different in middle BMI group compared to other groups
Lafortuna et al. (2008)	21 females BMI: 19.7 ± 1.1 kg/m^2^ (*n* = 6) BMI: 41.1 ± 5.0 kg/m^2^ (*n* = 15)	1.0 m/s	Not reported	Net W/kg not different between groups
LeCheminant et al. (2009)	26 females BMI: 22.2 ± 2.0 kg/m^2^ (*n* = 13) BMI: 27.2 ± 2.1 kg/m^2^ (*n* = 13)	1.34 m/s	Not reported	Net kJ/kg/min not different between groups

Several factors could potentially explain the conflicting findings. Overall, sample sizes were relatively small. Five of the 12 studies cited above had ≤10 participants in one or more of their BMI‐defined groups (Bode et al., 2020; Browning & Kram, 2005; Farrell et al., 1985; Lafortuna et al., 2008; Peyrot et al., 2009), and only four of the studies had >15 participants in any of their BMI‐defined groups (Browning et al., 2006, 2013; Peyrot et al., 2009; Primavesi et al., 2021). Due to the large individual variability in energy cost of walking (Gaesser et al., 2018), and the wide range in mean net Cw (1.71 to 2.45 J/kg/m) reported in the literature (Rubenson et al., 2007), a small sample size could complicate the interpretation and comparison of results and limit the generalizability of the findings. Also, not all studies confirmed whether steady‐state V̇O_2_ was established during walking trials (Table 1). Tolerable exercise intensities even slightly above the lactate threshold can elicit a slow component of V̇O_2_ (Poole et al., 1994), which delays attainment of steady‐state, and would have the effect of increasing the calculated Cw. Since individuals with obesity have a lower relative maximum oxygen uptake (V̇O_2max_) (Browning et al., 2006, 2013; Farrell et al., 1985; LeCheminant et al., 2009), exercise at a fixed walking speed elicits a higher percentage of their V̇O_2max_, which, if above the lactate threshold, might evoke a slow component of V̇O_2_ that would result in higher Cw. Finally, body mass‐adjusted standing metabolic rate (SMR) is also lower in individuals with obesity (Browning et al., 2006, 2013; Browning & Kram, 2005; Fernandez Menendez et al., 2020; Lafortuna et al., 2008; Primavesi et al., 2021). This can affect the interpretation of the data on the metabolic cost of walking (Browning et al., 2013; Browning & Kram, 2005; Fernandez Menendez et al., 2019). For example, Browning et al. (2013) reported that gross W/kg during walking was lower in persons with obesity but, due to a lower SMR, net W/kg was not different compared to persons without obesity.

Considering inconsistencies in previous literature due to small sample sizes, potential contributions of the slow component to energy expenditure during walking, and lack of control for SMR, the relationship between walking economy and measures of obesity remains unclear. Therefore, the purpose of our study was to determine whether body fat (total fat mass or body fat percentage) and BMI influenced net and gross Cw during moderate‐intensity walking (1.34 m/s) in a large sample of males and females with a wide range of body fat percentages and BMI, using energy expenditure data that were confirmed to be steady‐state.

## 2 MATERIALS AND METHODS

This study was approved by the Arizona State University Institutional Review Board and conformed to the ethical standards of the Declaration of Helsinki. A total of 230 healthy, nonsmoking adults, ages 18–81 years, were enrolled in this study. All participants provided written informed consent prior to participation. Participants were part of an NIH‐funded study that required energy expenditure measurements while performing a variety of physical activities. The sample size was dictated by the NIH grant and not for the specific purposes of the present secondary data analyses.

Complete details of the procedures have been described previously (Gaesser et al., 2018). Briefly, participants were instructed to consume nothing but water for 3 h immediately prior to arriving at the laboratory. Height was measured with a wall‐mounted stadiometer (Seca, Hamburg, Germany). Body weight, body fat percentage, fat mass, and fat‐free mass (FFM) were determined using a calibrated air displacement plethysmograph (BOD POD, COSMED, Rome, Italy). After height and body composition were assessed, participants were fitted with a lightweight, portable metabolic measurement system (Oxycon Mobile, Vyaire Medical, Yorba Linda, CA, USA) that has been validated against the Douglas Bag Method (Rosdahl et al., 2010). Calibration was performed before each testing visit according to the manufacturer's specifications.

As part of a protocol for our NIH‐sponsored study that required measurement of energy expenditure, all participants performed a 90‐min physical activity routine consisting of mostly light‐, and moderate‐intensity activities, one of which was walking on a treadmill (Trackmaster TMX 425, Full Vision Inc., Newton, KS) at 1.34 m/s (3.0 mph) for 8 min (Bhammar et al., 2016). Participants were not permitted to hold the handrails. Each activity was performed for 8 min, with 4 min of seated rest in between. Prior to the 90‐min routine, participants sat quietly for 5 min followed by 5 min of motionless standing.

Pulmonary ventilation and gas exchange were measured breath‐by‐breath continuously during rest and physical activity. V̇O_2_ and carbon dioxide production (V̇CO_2_) during the last 5 min of the walking bout, and the final 2 min of standing, were used to calculate gross and net metabolic rate (W/kg) (Gaesser et al., 2018; Lusk, 1924). Gross and net energy cost per meter (Cw; J/kg/m) were also calculated by dividing corresponding metabolic rate (W/kg) by walking speed (1.34 m/s).

### 2.1 Statistical analysis

All analyses were performed using SPSS 28.0 (IBM, Armonk, NY) with significance set at *p* < 0.05. All variables were presented as mean ± SD, unless stated otherwise. To ensure that only steady‐state V̇O_2_ was used to calculate metabolic rate, we eliminated any participant's data that exhibited a V̇O_2_ slow component (Poole et al., 1994) from the final analysis, which was defined as a significant (*p* < 0.05) positive beta coefficient (*β* > 0) from linear regression of V̇O_2_ versus time during the last 5 min of each 8‐min walking bout (Gaesser et al., 2018). Linear regression was used to assess relationships between body composition indices (body fat %, fat mass, BMI, and fat‐free mass) and both SMR and Cw. Unpaired *t*‐tests were used to assess differences between males and females. In addition, one‐way ANOVA was used to assess differences in both SMR and Cw across three BMI categories: normal weight (BMI < 25 kg/m^2^), overweight (BMI = 25.0–29.9 kg/m^2^), and obese (BMI ≥ 30 kg/m^2^). The Bonferroni correction was used for post hoc tests when appropriate.

## 3 RESULTS

Data from 13 participants were eliminated from data analyses due to a V̇O_2_ slow component, and data from 12 participants could not be used due to incomplete or missing data or equipment malfunction. Participant characteristics and metabolic responses during walking for the 13 participants eliminated from the final data analyses due to V̇O_2_ slow component are presented in Tables S1 and S2. Exclusion of these 13 participants did not change the primary outcomes of the study (Table S3). A total of 205 participants (115 females) were included in the final data analyses (Table 2). There were 102 participants with BMI < 25.0 kg/m^2^, 71 with BMI 25.0–29.9 kg/m^2^, and 32 with BMI ≥30 kg/m^2^. Three participants had BMIs <18.5 kg/m^2^, the lower limit for the normal weight BMI category. We confirmed that the results of the statistical analyses were unchanged after excluding these participants, all with BMIs between 17.5 and 18.4 kg/m^2^ (Table S4). Consequently, we included them in all data analyses.

**TABLE 2:** Participant characteristics (*n* = 205).

	All subjects	Male (*n* = 90)	Female (*n* = 115)
Age (years)	47 ± 21 (18–81)	49 ± 22 (18–78)	45 ± 20 (18–81)
Height (cm)	169.2 ± 9.4 (149.7–202.8)	176.6 ± 7.7 (160.5–202.8)	163.5 ± 6.0 (149.7–181.0)
Weight (kg)	73.6 ± 16.4 (45.4–136.2)	83.0 ± 14.7 (52.0–136.8)	66.2 ± 13.7 (45.4–110)
Body Mass Index (BMI) (kg/m^2^)	25.7 ± 4.7 (17.5–43.2)	26.6 ± 4.2 (19.3–43.2)	24.8 ± 4.9 (17.5–40.4)
BMI Classification (*n*, % of sample)			
BMI <25.0 kg/m^2^	102 (49.8%)	33 (36.7%)	69 (60.0%)
BMI 25.0–29.9 kg/m^2^	71 (34.6)	40 (44.4%)	31 (27.0%)
BMI ≥30.0 kg/m^2^	32 (15.6%)	17 (18.9%)	15 (13.0%)
Body fat percent (%)	28.3 ± 10.6 (3.0–52.8)	23.1 ± 9.9 (3.0–48.6)	32.3 ± 9.3 (11.4–52.8)
Fat mass (kg)	21.4 ± 11.0 (2.1–66.2)	20.1 ± 11.4 (2.1–66.2)	22.4 ± 10.6 (5.2–52.0)
Fat‐free mass (kg)	52.2 ± 11.7 (33.7–83.7)	62.9 ± 8.4 (49.0–83.7)	43.8 ± 5.2 (33.7–58.0)

*Note*: Data presented as Mean ± SD with minimum and maximum range in parentheses unless otherwise indicated.

**FIGURE 1:**
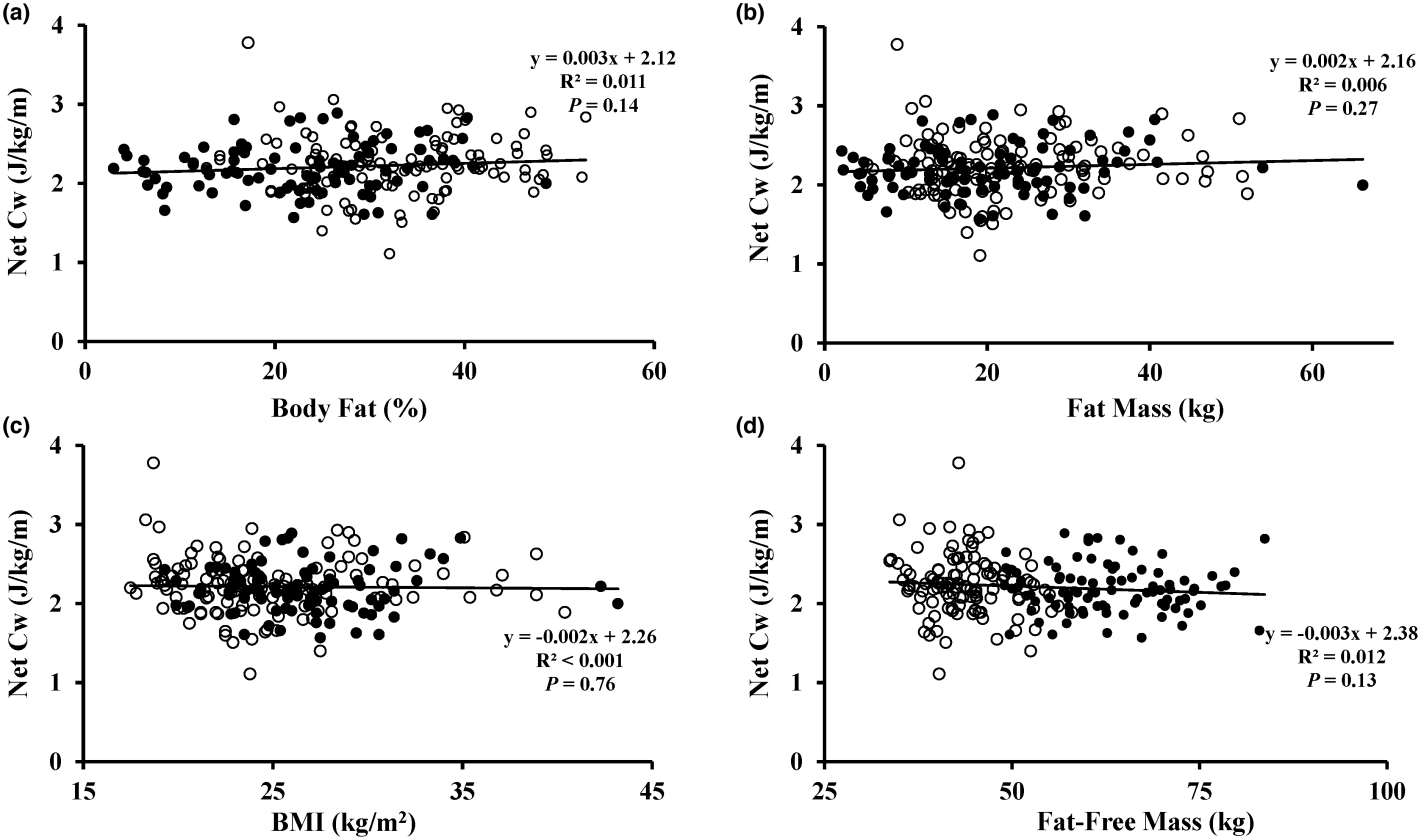
Net metabolic cost (Cw) (J/kg/m) during walking at 1.34 m/s versus percent body fat (a), body fat mass (b), body mass index (BMI) (c), and fat‐free mass (d). *n* = 205. Filled circles indicate males and open circles indicate females.

Net Cw was not related to percent body fat (*R* ^2^ = 0.011; *p* = 0.14), fat mass (*R* ^2^ = 0.006; *p* = 0.27), BMI (*R* ^2^ < 0.001; *p* = 0.76), or fat‐free mass (*R* ^2^ = 0.012; *p* = 0.13) (Figure 1a–d). Because net Cw is higher in participants ≥70 years (Gaesser et al., 2018), we also performed separate analyses split by age. The results were similar, as net Cw was not related to percent body fat, fat mass, BMI, or fat‐free mass in participants <70 years (*n* = 176) or ≥ 70 years (*n* = 29) (all *R* ^2^ < 0.076; all *p* > 0.05).

**FIGURE 2:**
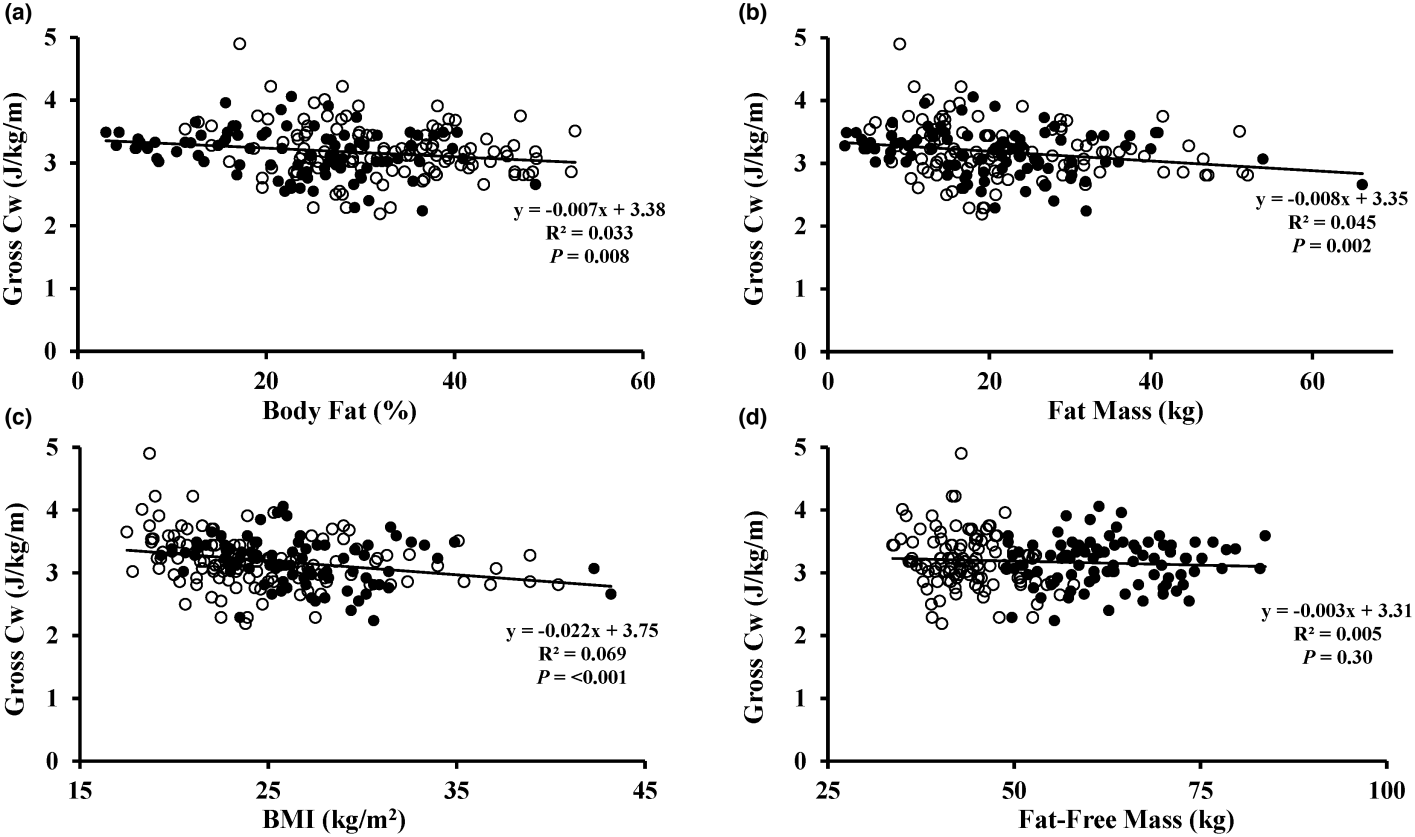
Gross metabolic cost (Cw) (J/kg/m) during walking at 1.34 m/s versus percent body fat (a), body fat mass (b), body mass index (BMI) (c), and fat‐free mass (d). *n* = 205. Filled circles indicate males and open circles indicate females.

**FIGURE 3:**
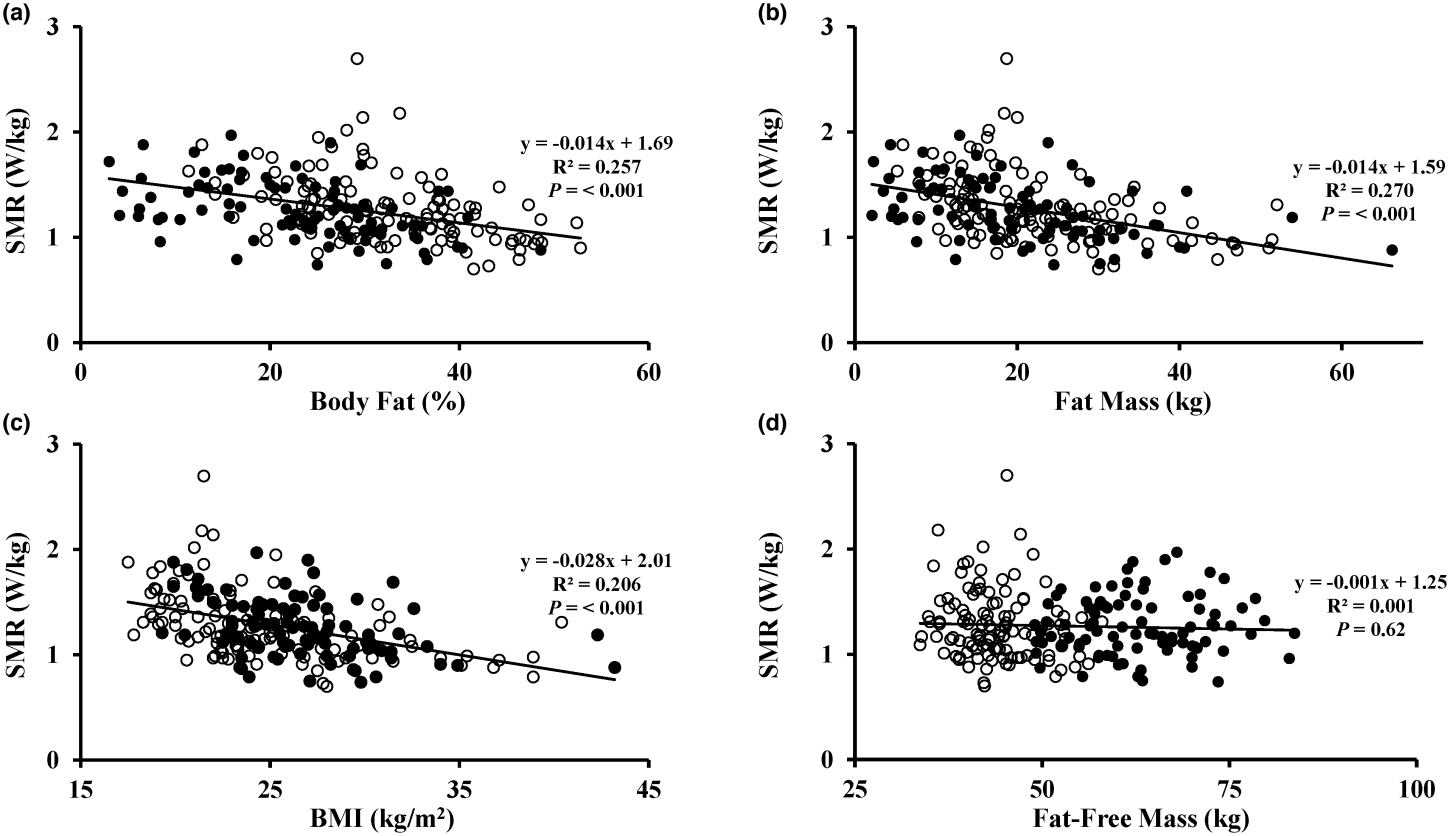
Standing metabolic rate (SMR) (W/kg) versus percent body fat (a), body fat mass (b), body mass index (BMI) (c), and fat‐free mass (d). *n* = 205. Filled circles indicate males and open circles indicate females.

Gross Cw was inversely related to percent body fat (*R* ^2^ = 0.033, *p* = 0.008), fat mass (*R* ^2^ = 0.045, *p* = 0.002), and BMI (*R* ^2^ = 0.069, *p* < 0.001) (Figure 2a–c). By contrast, gross Cw was not related to FFM (*R* ^2^ = 0.005, *p* = 0.30) (Figure 2d). SMR was inversely related to percent body fat (*R* ^2^ = 0.257, *p* < 0.001), fat mass (*R* ^2^ = 0.270, *p* < 0.001), and BMI (*R* ^2^ = 0.206, *p* < 0.001) (Figure 3a–c). By contrast, SMR was not related to FFM (*R* ^2^ = 0.001, *p* = 0.62) (Figure 3d).

No measure of metabolic rate (V̇O_2_, W/kg) or Cw (J/kg/m) differed by sex (Table 3). RER was lower in females (0.84 ± 0.05) than males (0.87 ± 0.05) during walking (*p* < 0.001). Gross metabolic rate (V̇O_2_, W/kg) and gross Cw were higher for participants with BMI < 25.0 kg/m^2^ compared to participants with BMI ≥ 25.0 kg/m^2^ (*p* = 0.01 gross V̇O_2_; *p* = 0.02 gross W/kg and gross Cw) (Table 4). Similarly, SMR (V̇O_2_, W/kg) was higher in individuals with BMI < 25.0 kg/m^2^ compared to individuals with BMI ≥ 25.0 kg/m^2^ (*p* < 0.001). However, no measure of net metabolic rate (V̇O_2_, W/kg) or Cw differed by BMI classification (Table 4).

**TABLE 3:** Standing and walking metabolic rate by sex (*n* = 205).

	All subjects	Male (*n* = 90)	Female (*n* = 115)	*p*
Standing				
V̇O_2_ (mL/kg/min)	3.8 ± 0.8	3.8 ± 0.8	3.8 ± 0.9	0.61
Respiratory exchange ratio (RER)	0.89 ± 0.06	0.90 ± 0.06	0.89 ± 0.06	0.40
Metabolic rate (W/kg)	1.30 ± 0.29	1.30 ± 0.28	1.28 ± 0.29	0.55
Walking at 1.34 m/s				
Gross V̇O_2_ (mL/kg/min)	12.6 ± 1.6	12.5 ± 1.5	12.6 ± 1.7	0.46
Respiratory exchange ratio (RER)	0.85 ± 0.06	0.87 ± 0.05	0.84 ± 0.05	<0.001
Net V̇O_2_ (mL/kg/min)	8.8 ± 1.4	8.7 ± 1.2	8.9 ± 1.5	0.23
Gross metabolic rate (W/kg)	4.26 ± 0.54	4.24 ± 0.49	4.28 ± 0.57	0.63
Net metabolic rate (W/kg)	2.97 ± 0.46	2.94 ± 0.40	3.00 ± 0.50	0.35
Gross energy cost/distance (J/kg/m)	3.18 ± 0.40	3.17 ± 0.36	3.19 ± 0.43	0.63
Net energy cost/distance (J/kg/m)	2.22 ± 0.34	2.19 ± 0.30	2.24 ± 0.37	0.35

*Note*: Data represent Mean ± SD. *p* values for between‐group comparisons for sex using unpaired *t*‐tests.

Abbreviation: V̇O_2_, oxygen uptake.

**TABLE 4:** Standing and walking metabolic rate by body mass index (BMI) classification (*n* = 205).

	Normal weight	Overweight	Obese	*p*
	BMI < 25 kg/m^2^ (*n* = 102)	BMI 25.0–29.9 kg/m^2^ (*n* = 71)	BMI ≥30 kg/m^2^ (*n* = 32)	
Standing				
V̇O_2_ (mL/kg/min)	4.1 ± 0.8*	3.7 ± 0.8	3.2 ± 0.6**	<0.001
Respiratory exchange ratio (RER)	0.89 ± 0.06	0.90 ± 0.07	0.89 ± 0.06	0.60
Metabolic rate (W/kg)	1.39 ± 0.28*	1.25 ± 0.28	1.10 ± 0.21**	<0.001
Walking at 1.34 m/s				
Gross V̇O_2_ (mL/kg/min)	12.9 ± 1.7*	12.3 ± 1.5	12.1 ± 1.4	0.01
Respiratory exchange ratio (RER)	0.85 ± 0.06	0.85 ± 0.05	0.87 ± 0.06	0.05
Net V̇O_2_ (mL/kg/min)	8.8 ± 1.4	8.6 ± 1.3	8.9 ± 1.2	0.60
Gross metabolic rate (W/kg)	4.37 ± 0.56	4.17 ± 0.51	4.12 ± 0.45	0.02
Net metabolic rate (W/kg)	2.98 ± 0.48	2.92 ± 0.45	3.02 ± 0.42	0.54
Gross energy cost/distance (J/kg/m)	3.26 ± 0.42	3.11 ± 0.38	3.08 ± 0.33	0.02
Net energy cost/distance (J/kg/m)	2.23 ± 0.36	2.18 ± 0.33	2.26 ± 0.31	0.54

*Note*: Data represent Mean ± SD. *p* values represent main effect comparisons by BMI class using One‐way ANOVA. *Denotes *p* < 0.05 difference for normal weight versus overweight and obese groups using Bonferroni post hoc test. **Denotes *p* < 0.001 difference for obese group versus normal weight group using Bonferroni post hoc test.

Abbreviation: V̇O_2_, oxygen uptake.

## 4 DISCUSSION

The results demonstrate that net Cw was unrelated to either body fat mass, percent body fat, BMI, or fat‐free mass in a large sample of males and females. The data displayed in Figure 1 illustrate that individuals with high body fat or high BMI do not have a greater net Cw during walking as compared to leaner individuals. The statistically significant inverse relationship between gross Cw and percent body fat, total fat mass, and BMI are in fact suggestive of modestly better walking economy in adults with high body fat content or BMI, although the low *R* ^2^ values indicate a lack of clinical utility.

Published data on the impact of BMI or body fat on the metabolic cost of walking are inconsistent. A higher net Cw or metabolic rate in adults with obesity has been reported in some studies (Browning et al., 2006; Browning & Kram, 2005; Fernandez Menendez et al., 2020; Primavesi et al., 2021) but not others (Bode et al., 2020; Browning et al., 2013; Fernandez Menendez et al., 2019; Lafortuna et al., 2008; LeCheminant et al., 2009). One study actually reported a lower relative V̇O_2_ (mL/kg/min) during walking in females with severe obesity compared to relatively lean females (Farrell et al., 1985). The inconsistencies in the published studies are observed even within laboratories. Browning and Kram (2005) initially reported that net W/kg across six walking speeds (0.50–1.75 m/s) was ~11% higher in females with a mean BMI of 34.1 kg/m^2^ compared to females with a mean BMI of 20.4 kg/m^2^, and then subsequently reported that both females and males with mean BMI of 33–34 kg/m^2^ had ~10% higher net W/kg compared to females and males with a mean BMI of ~20–22 kg/m^2^ (Browning et al., 2006). By contrast, Browning et al. (2013) later reported that net W/kg during walking at 1.25, 1.50, and 1.75 m/s was not different in females and males with similar differences in mean BMI as in their earlier studies (33.9 kg/m^2^ vs. 21.6 kg/m^2^). It is also important to note that in the first two studies, in which net W/kg was higher in participants with the higher BMI, gross Cw and gross V̇O_2_ (mL/kg/min) (i.e., without subtracting standing resting metabolic rate) were not different between groups (Browning et al., 2006; Browning & Kram, 2005). This latter finding is due to the fact that the standing metabolic rate is inversely related to BMI. The authors also suggested that the inconsistent findings between studies could be due in large part to the inherent between‐subject variability in walking and standing metabolic rates (Rubenson et al., 2007) (further discussion below).

Fernandez Menendez et al. (2020) reported in one study that net Cw was ~10%–45% higher in adults with higher BMI (mean 43.0 kg/m^2^ vs. 22.0 kg/m^2^) at all five walking speeds studied (0.56–1.67 m/s) (Fernandez Menendez et al., 2020), yet an earlier study (Fernandez Menendez et al., 2019) by the same group found that net Cw was not influenced by BMI status. In the earlier study (Fernandez Menendez et al., 2019), net Cw was not different between groups with different mean BMI (33.8 kg/m^2^ vs. 21.9 kg/m^2^) at the four slowest speeds, but was only significantly greater in the higher‐BMI group at the fastest walking speed (1.67 m/s). A third study (Primavesi et al., 2021) from this group, that used pooled data from the first two studies along with additional participants, found that net Cw in individuals with grade II or III obesity (BMI ≥ 35 m/kg^2^; mean = 40.1 m/kg^2^) was 15% higher than net Cw in individuals with a BMI in the normal‐weight range (BMI 18.5–24.9 kg/m^2^; mean = 22.0 m/kg^2^) but nonsignificantly 6% higher than individuals with grade I obesity (BMI 30–34.9 m/kg^2^; mean = 32.2 kg/m^2^). The authors posited that the degree of obesity influences net Cw. However, a BMI >35 kg/m^2^ is not always associated with higher walking energy expenditure. For example, Farrell et al. (1985) reported that females with a mean BMI 48.0 kg/m^2^ (50.5% body fat) had a lower V̇O_2_ (mL/kg/min) during walking at 1.25 m/s compared to females with 23.4% body fat (BMI 22.2 kg/m^2^). Also, Browning et al. (2006) reported that across a range of walking speeds, individuals with grade III obesity (BMI > 40.0 kg/m^2^) had similar net metabolic rate and net Cw as females with class II obesity (BMI 35.0–39.9 kg/m^2^) and males with a mean BMI of 22.3 m/kg^2^.

Lastly, the divergent findings from Laroche and colleagues are worth noting (Bode et al., 2020; Laroche et al., 2015). Among older adults, gross Cw (resting metabolic rate not reported) during walking at 0.83 m/s was 20% higher in participants with a mean BMI of 29.5 kg/m^2^ compared to participants with a mean BMI of 22.4 kg/m^2^ (Laroche et al., 2015). In a subsequent paper (Bode et al., 2020), both gross and net Cw walking at 1.25 m/s were not different in participants with mean BMI of 33.1 kg/m^2^ versus 22.4 kg/m^2^, even though the difference in BMI between groups was greater in the second study. Most surprising is that in the first study by Laroche et al. (2015) gross Cw was positively correlated with % body fat (*r* = 0.42; *p* = 0.008) and fat mass (*r* = 0.58; *p* = 0.001), whereas in the second study by Bode et al. (2020) gross Cw was *inversely* correlated with BMI (*r* = −0.44; *p* = 0.008).

Small sample sizes and methodological issues, such as not verifying steady‐state V̇O_2_ during the walking sessions (Table 1), may have contributed to the inconsistent results. The issue of small sample size is especially important considering the large variation in Cw across individuals, as evident in Figures 1 and 2. Except for a few outliers, individual net Cw ranged between ~1.5 and 3.0 J/kg/m. Ten of the 12 studies cited above included only 6–13 participants in at least one of their groups (Bode et al., 2020; Browning & Kram, 2005; Farrell et al., 1985; Fernandez Menendez et al., 2019, 2020; Lafortuna et al., 2008; Laroche et al., 2015; LeCheminant et al., 2009; Peyrot et al., 2009; Primavesi et al., 2021). The combination of small sample size and large individual variability in net J/kg/m (Gaesser et al., 2018) would increase the likelihood of discrepant results among studies. The literature indicates considerable variability in net Cw, with mean values in various studies ranging from 1.71 to 2.45 J/kg/m, with an overall mean of 2.05 J/kg/m based on a review of 20 studies (Rubenson et al., 2007). This represents a 43% difference between the high and low values. Our overall mean of 2.22 J/kg/m (Table 3) is close to the overall mean from these studies, and likely reflects our large sample of 205 adults.

Net metabolic rate (Browning et al., 2006) and gross Cw (Laroche et al., 2015) have been reported to be positively correlated with percent body fat, and gross Cw has been reported to be positively correlated with BMI (Laroche et al., 2015). Our results challenge these findings, and suggest that the positive correlations may be spurious, perhaps due to small sample sizes. Indeed, small sample sizes (20–26 total participants) may explain the puzzling findings from the same laboratory that gross Cw was both positively (Laroche et al., 2015) and inversely (Bode et al., 2020) correlated with BMI.

It is known that older adults (≥ 70 years) have a higher energy cost of walking (Gaesser et al., 2018). To investigate whether age affects the relationship between net Cw and percent body fat, we performed separate analyses for participants <70 years and ≥70 years. For both age ranges, net Cw was not correlated with percent body fat. Similar results were observed for total body fat and BMI. Collectively, our data strongly suggest that percent body fat does not influence net Cw. Figure 1 also illustrates that BMI, ranging from 17.5 to 43.2 kg/m^2^, is unrelated to net Cw.

Our finding that body fat and BMI were not correlated with net Cw suggests that the efficiency of muscular work is similar in individuals with and without obesity. Cycle ergometer work efficiency (defined as the inverse of the slope of the V̇O_2_‐work rate relationship) has been shown to be unrelated to BMI across a BMI range of ~20–50 kg/m^2^ (Lafortuna et al., 2008), and both work and delta efficiency (defined as the change in energy expenditure for a given change in work rate (Gaesser & Brooks, 1975)) have been reported to be unrelated to body mass in adults weighing between 40 and 100 kg (Berry et al., 1993). Also, we performed additional analyses on data from 168 participants in our current study who also performed cycle ergometer exercise as previously reported (Gaesser et al., 2018), and found that net cycling efficiency was not significantly correlated with percent body fat (*r* = 0.07; *p* = 0.38) or BMI (*r* = −0.13; *p* = 0.11). If cycle ergometry can be assumed to be a better mode of exercise for assessment of muscular efficiency (Lafortuna et al., 2008), these data suggest that apparent efficiency of muscle energy transduction is not affected by obesity.

Gross Cw was inversely related to body fat and BMI, although the statistical significance is due primarily to the large sample size and not the strength of the regression. We hesitate to place much significance on this finding in view of the very low percentage of variance in Cw explained by either body fat or BMI. However, these results provide additional support to our primary conclusion that energy expenditure during walking is not higher in individuals with obesity compared to individuals without obesity. Moreover, our data emphasize the importance of distinguishing between net and gross metabolic rate for the interpretation of the energy cost of walking. The lower gross Cw for individuals with higher body fat or BMI is consistent with our observation that SMR was also significantly inversely correlated with body fat and BMI and is in line with previous reports (Browning et al., 2006, 2013; Browning & Kram, 2005; Fernandez Menendez et al., 2020; Lafortuna et al., 2008; Peyrot et al., 2009; Primavesi et al., 2021). The inverse relationship between both SMR and gross Cw with both percent body fat and BMI explains why net Cw is unrelated to body fat and BMI (i.e., a lower gross Cw is offset by a smaller correction for SMR).

### 4.1 Strengths and limitations

Our study had several strengths. The sample size of 205 males and females is considerably larger than that of previous studies that have investigated the relationship between BMI, body fat, and the energy cost of walking in adults. This is important due to the large individual variation in energy expenditure during walking. Our sample included a wide range of BMI (17.5 kg/m^2^ to 43.2 kg/m^2^) and percent body fat (3.0% fat to 52.8% fat). In a subset of 43 participants included in this study, we previously reported a high test–retest reliability for gross and net kcal/kg/min and J/kg/m during treadmill walking, with all intraclass correlation coefficients between 0.80 and 0.86 (Gaesser et al., 2018). Thus, we have confidence in our results, which also indicate that the considerable between‐subject variation in J/kg/m during walking evident in Figures 1 and 2 is reproducible. We also removed from data analysis any participant for whom a V̇O_2_ slow component was detected during the 8‐min walking trial, thus ensuring steady‐state V̇O_2_ for all calculations. Verification of steady‐state has not always been rigorously documented in previous studies (Table 1).

Our study also had some limitations. We determined energy expenditure walking at only one speed. However, the selected walking speed of 1.34 m/s is very close to the walking speed associated with the minimum energy cost per distance in adults with and without obesity (Browning et al., 2006; Browning & Kram, 2005) and in older adults (Martin et al., 1992). It is also within the range of preferred walking speeds reported by others (~0.75 m/s to 1.47 m/s), which varies according to age and degree of obesity (Browning et al., 2006; Browning & Kram, 2005; Malatesta et al., 2009; Mattsson et al., 1997; Ohrstrom et al., 2001; Spyropoulos et al., 1991). Our selected speed of 1.34 is also similar to that used by others (Browning et al., 2006, 2013; Browning & Kram, 2005; LeCheminant et al., 2009). It is possible that obesity may have a greater impact on Cw at higher walking speeds (Browning et al., 2006; Browning & Kram, 2005; Fernandez Menendez et al., 2019, 2020; Primavesi et al., 2021), although the largest previous study to address this reported that net W/kg was not affected by BMI at speeds up to 1.75 m/s (Browning et al., 2013). Having participants consume nothing but water for the 3‐h period prior to arriving at the laboratory may not have been sufficient to completely eliminate the effect of dietary‐induced thermogenesis resulting from food or caffeine ingestion prior to that time (Reed & Hill, 1996; Segal et al., 1985). However, measurements of resting and exercise EE were not taken until approximately 1 h after arrival at the laboratory, and postprandial elevation of metabolic rate is largely over by ~4 h (Reed & Hill, 1996; Segal et al., 1985). Caffeine consumption before the 3‐h period prior to arrival at the laboratory could have affected resting metabolic rate, but caffeine has been reported to have little to no effect on exercise metabolic rate (Bracco et al., 1995).

## 5 CONCLUSION

Our results demonstrate that net Cw in adults during treadmill walking at 1.34 m/s in a laboratory setting is not influenced by body fat percentage, total body fat mass, and BMI. Furthermore, net Cw and metabolic rate did not differ by sex or across different BMI categories. Gross Cw and SMR are weakly and moderately inversely related to body fat percentage, total body fat, and BMI, respectively. Higher body fat does not appear to reduce walking economy in adults.

## AUTHOR CONTRIBUTIONS

G.A.G. conceptualized and designed the study. W.J.T., B.J.S., D.M.B., and S.S.A. were responsible for data collection. W.J.T. and E.W.W. performed the statistical analysis. W.J.T., B.J.S., D.M.B., E.W.W., and S.S.A. interpreted the data and prepared the results for publication. W.J.T. and G.A.G. wrote the manuscript. All authors reviewed and edited the manuscript and approved the final version for publication.

## FUNDING INFORMATION

Research funded by NIH National Heart, Lung, and Blood Institute Grant R01‐HL‐091006.

## CONFLICT OF INTEREST STATEMENT

No conflicts of interest, financial or otherwise, are declared by the authors.

## 6 ETHICS STATEMENT

This study was approved by the Arizona State University Institutional Review Board and conformed to the ethical standards of the Declaration of Helsinki.

## Supporting information


Tables S1–S4.

## Data Availability

Data available from the corresponding author upon request.

## References

[phy216023-bib-0001] Berry, M. J. , Storsteen, J. A. , & Woodard, C. M. (1993). Effects of body mass on exercise efficiency and VO2 during steady‐state cycling. Medicine and Science in Sports and Exercise, 25, 1031–1037. 8231771

[phy216023-bib-0002] Bhammar, D. M. , Sawyer, B. J. , Tucker, W. J. , Lee, J. M. , & Gaesser, G. A. (2016). Validity of SenseWear(R) armband v5.2 and v2.2 for estimating energy expenditure. Journal of Sports Sciences, 34, 1830–1838. 26854829 10.1080/02640414.2016.1140220 PMC5047752

[phy216023-bib-0003] Bode, V. G. , Croce, R. V. , Quinn, T. J. , & Laroche, D. P. (2020). Influence of excess weight on lower‐extremity vertical stiffness and metabolic cost of walking. European Journal of Sport Science, 20, 477–485. 31405356 10.1080/17461391.2019.1652350

[phy216023-bib-0004] Bracco, D. , Ferrarra, J. M. , Arnaud, M. J. , Jequier, E. , & Schutz, Y. (1995). Effects of caffeine on energy metabolism, heart rate, and methylxanthine metabolism in lean and obese women. The American Journal of Physiology, 269, E671–E678. 7485480 10.1152/ajpendo.1995.269.4.E671

[phy216023-bib-0005] Browning, R. C. , Baker, E. A. , Herron, J. A. , & Kram, R. (2006). Effects of obesity and sex on the energetic cost and preferred speed of walking. Journal of Applied Physiology (1985), 100, 390–398. 10.1152/japplphysiol.00767.2005 16210434

[phy216023-bib-0006] Browning, R. C. , & Kram, R. (2005). Energetic cost and preferred speed of walking in obese vs. normal weight women. Obesity Research, 13, 891–899. 15919843 10.1038/oby.2005.103

[phy216023-bib-0007] Browning, R. C. , Reynolds, M. M. , Board, W. J. , Walters, K. A. , & Reiser, R. F., 2nd . (2013). Obesity does not impair walking economy across a range of speeds and grades. Journal of Applied Physiology (1985), 114, 1125–1131. 10.1152/japplphysiol.00765.2012 PMC3656435 23412900

[phy216023-bib-0008] Farrell, P. A. , Gustafson, A. B. , & Kalkhoff, R. K. (1985). Assessment of methods for assigning treadmill exercise workloads for lean and obese women. International Journal of Obesity, 9, 49–58. 4019018

[phy216023-bib-0009] Fernandez Menendez, A. , Saubade, M. , Millet, G. P. , & Malatesta, D. (2019). Energy‐saving walking mechanisms in obese adults. Journal of Applied Physiology (1985), 126, 1250–1258. 10.1152/japplphysiol.00473.2018 30817245

[phy216023-bib-0010] Fernandez Menendez, A. , Uva, B. , Favre, L. , Hans, D. , Borrani, F. , & Malatesta, D. (2020). Mass‐normalized internal mechanical work in walking is not impaired in adults with class III obesity. Journal of Applied Physiology (1985), 129, 194–203. 10.1152/japplphysiol.00837.2019 32584667

[phy216023-bib-0011] Gaesser, G. A. , & Brooks, G. A. (1975). Muscular efficiency during steady‐rate exercise: Effects of speed and work rate. Journal of Applied Physiology, 38, 1132–1139. 1141128 10.1152/jappl.1975.38.6.1132

[phy216023-bib-0012] Gaesser, G. A. , Tucker, W. J. , Sawyer, B. J. , Bhammar, D. M. , & Angadi, S. S. (2018). Cycling efficiency and energy cost of walking in young and older adults. Journal of Applied Physiology (1985), 124, 414–420. 10.1152/japplphysiol.00789.2017 PMC5867372 29146688

[phy216023-bib-0013] James, W. P. , Davies, H. L. , Bailes, J. , & Dauncey, M. J. (1978). Elevated metabolic rates in obesity. Lancet, 1, 1122–1125. 77416 10.1016/s0140-6736(78)90300-8

[phy216023-bib-0014] Lafortuna, C. L. , Agosti, F. , Galli, R. , Busti, C. , Lazzer, S. , & Sartorio, A. (2008). The energetic and cardiovascular response to treadmill walking and cycle ergometer exercise in obese women. European Journal of Applied Physiology, 103, 707–717. 18496708 10.1007/s00421-008-0758-y

[phy216023-bib-0015] Laroche, D. P. , Marques, N. R. , Shumila, H. N. , Logan, C. R. , Laurent, R. S. , & Goncalves, M. (2015). Excess body weight and gait influence energy cost of walking in older adults. Medicine and Science in Sports and Exercise, 47, 1017–1025. 25202852 10.1249/MSS.0000000000000501 PMC4362814

[phy216023-bib-0016] LeCheminant, J. D. , Heden, T. , Smith, J. , & Covington, N. K. (2009). Comparison of energy expenditure, economy, and pedometer counts between normal weight and overweight or obese women during a walking and jogging activity. European Journal of Applied Physiology, 106, 675–682. 19408007 10.1007/s00421-009-1059-9

[phy216023-bib-0017] Lusk, G. (1924). Animal calorimetry twenty‐fourth paper. Analysis of the oxidation of mixtures of carbohydrate and fat. The Journal of Biological Chemistry, 59, 41–42.

[phy216023-bib-0018] Malatesta, D. , Vismara, L. , Menegoni, F. , Galli, M. , Romei, M. , & Capodaglio, P. (2009). Mechanical external work and recovery at preferred walking speed in obese subjects. Medicine and Science in Sports and Exercise, 41, 426–434. 19127181 10.1249/MSS.0b013e31818606e7

[phy216023-bib-0019] Martin, P. E. , Rothstein, D. E. , & Larish, D. D. (1992). Effects of age and physical activity status on the speed‐aerobic demand relationship of walking. Journal of Applied Physiology (1985), 73, 200–206. 10.1152/jappl.1992.73.1.200 1506370

[phy216023-bib-0020] Mattsson, E. , Larsson, U. E. , & Rossner, S. (1997). Is walking for exercise too exhausting for obese women? International Journal of Obesity and Related Metabolic Disorders, 21, 380–386. 9152740 10.1038/sj.ijo.0800417

[phy216023-bib-0021] Ohrstrom, M. , Hedenbro, J. , & Ekelund, M. (2001). Energy expenditure during treadmill walking before and after vertical banded gastroplasty: A one‐year follow‐up study in 11 obese women. The European Journal of Surgery, 167, 845–850. 11848239 10.1080/11024150152717689

[phy216023-bib-0022] Peyrot, N. , Thivel, D. , Isacco, L. , Morin, J. B. , Duche, P. , & Belli, A. (2009). Do mechanical gait parameters explain the higher metabolic cost of walking in obese adolescents? Journal of Applied Physiology (1985), 106, 1763–1770. 10.1152/japplphysiol.91240.2008 19246657

[phy216023-bib-0023] Poole, D. C. , Barstow, T. J. , Gaesser, G. A. , Willis, W. T. , & Whipp, B. J. (1994). VO2 slow component: Physiological and functional significance. Medicine and Science in Sports and Exercise, 26, 1354–1358. 7837956

[phy216023-bib-0024] Primavesi, J. , Fernandez Menendez, A. , Hans, D. , Favre, L. , Crettaz von Roten, F. , & Malatesta, D. (2021). The effect of obesity class on the energetics and mechanics of walking. Nutrients, 13, 4546. 34960097 10.3390/nu13124546 PMC8703877

[phy216023-bib-0025] Reed, G. W. , & Hill, J. O. (1996). Measuring the thermic effect of food. The American Journal of Clinical Nutrition, 63, 164–169. 8561055 10.1093/ajcn/63.2.164

[phy216023-bib-0026] Rosdahl, H. , Gullstrand, L. , Salier‐Eriksson, J. , Johansson, P. , & Schantz, P. (2010). Evaluation of the Oxycon Mobile metabolic system against the Douglas bag method. European Journal of Applied Physiology, 109, 159–171. 20043228 10.1007/s00421-009-1326-9

[phy216023-bib-0027] Rubenson, J. , Heliams, D. B. , Maloney, S. K. , Withers, P. C. , Lloyd, D. G. , & Fournier, P. A. (2007). Reappraisal of the comparative cost of human locomotion using gait‐specific allometric analyses. The Journal of Experimental Biology, 210, 3513–3524. 17921153 10.1242/jeb.000992

[phy216023-bib-0028] Segal, K. R. , Gutin, B. , Nyman, A. M. , & Pi‐Sunyer, F. X. (1985). Thermic effect of food at rest, during exercise, and after exercise in lean and obese men of similar body weight. The Journal of Clinical Investigation, 76, 1107–1112. 4044828 10.1172/JCI112065 PMC424000

[phy216023-bib-0029] Spyropoulos, P. , Pisciotta, J. C. , Pavlou, K. N. , Cairns, M. A. , & Simon, S. R. (1991). Biomechanical gait analysis in obese men. Archives of Physical Medicine and Rehabilitation, 72, 1065–1070. 1741658

