# Supplementary material for: Treadmill walking economy is not affected by body fat and body mass index in adults
Source: Physiol Rep. 2024 May 17;12(10):e16023. doi: 10.14814/phy2.16023 (PMC11101323; doi:10.14814/phy2.16023)
Supplement: Supplementary file 1 — Tables S1–S4. [file PHY2-12-e16023-s001.docx]

**Supplementary Table 1.** Characteristics of participants excluded from final analyses due to V̇O_2_ slow component (n = 13).

**Variable Mean ± SD (Min – Max)**

| Age (years) | 53 ± 20 (21 - 74) |
| --- | --- |
| Female (n, % of sample) | 7 (53.8%) |
| Height (cm) | 170.0 ± 8.6 (155.0 – 186.5) |
| Weight (kg) | 81.8 ± 17.8 (57.3 – 107.4) |
| Body Mass Index (BMI) (kg/m^2^) | 28.2 ± 5.6 (20.4 – 36.0) |
| BMI Classification (n, % of sample) |  |
| BMI < 25.0 kg/m^2^ | 5 (38.5%) |
| BMI 25.0 – 29.9 kg/m^2^ | 3 (23.1%) |
| BMI ≥ 30.0 kg/m^2^ | 5 (38.5%) |
| Body fat percent (%) | 34.6 ± 9.2 (18.8 – 47.0) |
| Fat mass (kg) | 29.3 ± 12.2 (10.8 – 46.6) |
| Fat-free mass (kg) | 52.5 ± 8.9 (40.8 – 66.3) |

* Data presented as Mean ± SD with minimum and maximum range in parentheses unless otherwise indicated.

**Supplementary Table 2.** Standing and walking metabolic rate in participants excluded from final analyses due to V̇O_2_ slow component (n = 13).

| **Variable** | **Mean ± SD (Min – Max)** |
| --- | --- |
| **Standing** |  |
| V̇O_2_ (ml/kg/min) | 3.6 ± 0.9 (2.2 – 5.6) |
| RER | 0.88 ± 0.06 (0.80 – 0.98) |
| Metabolic Rate (W/kg) | 1.22 ± 0.31 |
| **Walking at 1.34 m/s** |  |
| Gross V̇O_2_ (ml/kg/min) | 12.4 ± 1.8 (7.8 – 14.5) |
| RER | 0.84 ± 0.07 (0.77 – 1.02) |
| Net V̇O_2_ (ml/kg/min) | 8.8 ± 1.5 (4.7 – 10.6) |
| Gross Metabolic Rate (W/kg) | 4.21 ± 0.59 (2.65 – 4.83) |
| Net Metabolic Rate (W/kg) | 2.99 ± 0.51 (1.62 – 3.59) |
| Gross Energy Cost/Distance (J/kg/m) | 3.15 ± 0.44 (1.98 – 3.60) |
| Net Energy Cost/Distance (J/kg/m) | 2.23 ± 0.38 (1.21 – 2.68) |

* Data presented as Mean ± SD with minimum and maximum range in parentheses unless otherwise indicated.

**Supplementary Table 3.** Linear regressions for relationship between body composition indices (body fat %, fat mass, body mass index [BMI], fat-free mass) and energy cost of walking (Cw) with inclusion of 13 participants who exhibited V̇O_2_ slow component during walking (n = 218) or exclusion of 13 participants who exhibited V̇O_2_ slow component during walking (n = 205).

| **Model** | **R^2^ values** | ***P* values** |
| --- | --- | --- |
| **Gross Energy Cost/Distance (J/kg/m) (n = 218)** | | |
| Body Fat (%) | 0.039 | 0.004 |
| Fat mass (kg) | 0.043 | 0.002 |
| BMI (kg/m^2^) | 0.061 | < 0.001 |
| Fat-free mass (kg) | 0.002 | 0.507 |
| **Net Energy Cost/Distance (J/kg/m) (n = 218)** | | |
| Body Fat (%) | 0.009 | 0.160 |
| Fat mass (kg) | 0.007 | 0.221 |
| BMI (kg/m^2^) | < 0.001 | 0.940 |
| Fat-free mass (kg) | 0.007 | 0.231 |
| **Gross Energy Cost/Distance (J/kg/m) (n = 205)** | | |
| Body Fat (%) | 0.033 | 0.008 |
| Fat mass (kg) | 0.045 | 0.002 |
| BMI (kg/m^2^) | 0.069 | < 0.001 |
| Fat-free mass (kg) | 0.005 | 0.30 |
| **Net Energy Cost/Distance (J/kg/m) (n = 205)** | | |
| Body Fat (%) | 0.011 | 0.014 |
| Fat mass (kg) | 0.006 | 0.27 |
| BMI (kg/m^2^) | < 0.001 | 0.76 |
| Fat-free mass (kg) | 0.012 | 0.13 |

**Supplementary Table 4.** Linear regressions for relationship between body composition indices (body fat %, fat mass, body mass index [BMI], fat-free mass) and energy cost of walking (Cw) with exclusion of 3 participants with BMI < 18.5 kg/m^2^ (n = 202) or inclusion of 3 participants with BMI < 18.5 kg/m^2^ (n = 205).

| **Model** | **R^2^ values** | ***P* values** |
| --- | --- | --- |
| **Gross Energy Cost/Distance (J/kg/m) (n = 202)** | | |
| Body Fat (%) | 0.032 | 0.011 |
| Fat mass (kg) | 0.041 | 0.004 |
| BMI (kg/m^2^) | 0.061 | < 0.001 |
| Fat-free mass (kg) | 0.003 | 0.432 |
| **Net Energy Cost/Distance (J/kg/m) (n = 202)** | | |
| Body Fat (%) | 0.011 | 0.131 |
| Fat mass (kg) | 0.008 | 0.214 |
| BMI (kg/m^2^) | < 0.001 | 0.941 |
| Fat-free mass (kg) | 0.009 | 0.187 |
| **Gross Energy Cost/Distance (J/kg/m) (n = 205)** | | |
| Body Fat (%) | 0.033 | 0.008 |
| Fat mass (kg) | 0.045 | 0.002 |
| BMI (kg/m^2^) | 0.069 | < 0.001 |
| Fat-free mass (kg) | 0.005 | 0.30 |
| **Net Energy Cost/Distance (J/kg/m) (n = 205)** | | |
| Body Fat (%) | 0.011 | 0.014 |
| Fat mass (kg) | 0.006 | 0.27 |
| BMI (kg/m^2^) | < 0.001 | 0.76 |
| Fat-free mass (kg) | 0.012 | 0.13 |
|  |  |  |
